# High-Conductive CsH_2_PO_4_ Membranes with PVDF-Based Polymers Additives

**DOI:** 10.3390/membranes13070617

**Published:** 2023-06-22

**Authors:** Irina Bagryantseva, Valentina Ponomareva, Yuri Kungurtsev

**Affiliations:** 1 Institute of Solid State Chemistry and Mechanochemistry SB RAS, 630090 Novosibirsk, Russia; 2 Department of Natural Sciences, Novosibirsk State University, 630090 Novosibirsk, Russia

**Keywords:** proton conductivity, polymer electrolyte membrane, CsH_2_PO_4_, fluoropolymer, solid acid fuel cell, thin-film membranes

## Abstract

The study is devoted to one of the important problems of hydrogen energy—the comparative analysis and creation of novel highly conductive and durable medium-temperature proton membranes based on cesium dihydrogen phosphate and fluoropolymers. The proton conductivity, structural characteristics and mechanical properties of (1 − x)CsH_2_PO_4_-x fluoropolymer electrolytes (x-mass fraction, x = 0–0.3) have been investigated and analyzed. UPTFE and PVDF-based polymers (F2M, F42, and SKF26) with high thermal stability and mechanical properties have been chosen as polymer additives. The used fluoropolymers are shown to be chemical inert matrices for CsH_2_PO_4_. According to the XRD data, a monoclinic CsH_2_PO_4_ (P2_1_/m) phase was retained in all of the polymer electrolytes studied. Highly conductive and mechanically strong composite membranes with thicknesses of ~50–100 μm were obtained for the soluble fluoropolymers (F2M, F42, and SKF26). The size and shape of CsH_2_PO_4_ particles and their distribution have been shown to significantly affect proton conductivity and the mechanical properties of the membranes. The thin-film polymer systems with uniform distributions of salt particles (up to ~300 nm) were produced via the use of different methods. The best results were achieved via the pretreatment of the suspension in a bead mill. The ability of the membranes to resist plastic deformation increases with the growth of the polymer content in comparison with the pure CsH_2_PO_4_, and the values of the mechanical strength characteristics are comparable to the best low-temperature polymer membranes. The proton-conducting membranes (1 − x)CsH_2_PO_4_-x fluoropolymer with the optimal combination of the conductivity and mechanical and hydrophobic properties are promising for use in solid acid fuel cells and other medium-temperature electrochemical devices.

## 1. Introduction

High-temperature superionic phases of acid salts of alkali metals are characterized by the structural disordering of hydrogen bond networks, resulting in the high mobility of protons and high conductivity values up to σ ~10^−3^ to 10^−2^ S/cm at 100–250 °C [1,2]. For CsH_2_PO_4_, the phase transition from monoclinic (P2_1_/m) to a cubic superionic (Pm-3m) phase with a conductivity of σ~6 × 10^−2^ S/cm is observed at 230 °C [3]. In the superionic disordered phase, all protons participate in the transfer process provided by tetrahedra reorientation followed by proton hopping [4,5]. Publications on acid salts of alkali metals have mainly focused on their use as electrolytes for medium-temperature fuel cells SAFC [6,7,8,9]. Recently, the possibility of using of acid salt based solid electrolytes in the process of water electrolysis has been shown for the medium-temperature range of 230–400 °C. A higher operating temperature in comparison with the low-temperature devices allows to improve the electrochemical performance and replace platinum catalysts [10,11,12]. Additionally, the medium-temperature range of 200–400 °C is suitable for the synthesis of various organic substances, such as methane and methanol. It was shown that, during the combined electrolysis of CO_2_ and H_2_O using a nickel cathode and an IrO_2_ anode, a CsH_2_PO_4_-SiC composite electrolyte (T = 300 °C, pressure 8 bar), it is possible to carry out the synthesis of methane. The maximum efficiency for methane synthesis was obtained at a current density of 10–15 mA/cm^2^ [13].

The development of flexible CsH_2_PO_4_ polymer films can significantly improve the disadvantageous properties of acid salt membranes, such as insufficient mechanical properties and water solubility. Additionally, the ohmic losses can be decreased by reducing the thickness of the hybrid membrane. Various polymers were considered earlier as effective additives for obtaining CsH_2_PO_4_-based membranes: SPEEK [14], Butvar [15], and epoxy resin [16,17]. The properties of such composite systems are combinations of strength, flexibility, and hydrophobicity in terms of polymer components and the high conductivity of inorganic CsH_2_PO_4_ salt. Butvar and epoxy resin are chemically inert to CsH_2_PO_4_ polymers and soluble in different solvents; therefore, it is possible to obtain thin-film flexible membranes. For SPEEK polymer composites, changes in the phase composition with the formation of CsH_5_(PO_4_)_2_ was observed [14]. Polymerized epoxy resin and polyvinyl butyral (Butvar) have high thermal stability with no weight loss or phase change when heated up to 290 °C and have high flexural strength values of 39.8 and 38.6 MPa, respectively. Polymers have negligible electrical conductivity in the operating temperature range. An optimal combination of high values for proton conductivity and high flexural strength (7.3 MPa) were obtained for (1 − x)CsH_2_PO_4_-x epoxy resin composite (x = 0.2) [16]. However, this system was characterized by average-sized salt particles of less than 10 μm were obtained via a prolonged (4 days) treatment in a ball mill that could influence the component distribution, the thickness of the membranes and gas permeability. The advantages of Butvar (PVB, polyvinyl butyral) are solubility in a wide range of solvents such as alcohols and good stability in acidic and basic media. The solubility of PVB in ethanol and isopropanol allows the synthesis of small CsH_2_PO_4_ particles from initial Cs_2_CO_3_ and H_3_PO_4_ (soluble in alcohols) directly in a polymer solution [15]. The limit of the thermal stability range of the studied polymers is close to the operating temperatures of CsH_2_PO_4_, while for the fluoropolymers, the thermal stability range is much higher.

Fluoropolymers (FPs) are an extensive class of functional materials with perfect thermal stability and chemical inertness, hydrophobic properties and the ability to operate in highly extreme conditions [18]. Additionally, they have been suggested for use as polymer additives for CsH_2_PO_4_-based membranes. Polymer additives makes cesium-based electrolyte more stable in terms of deformation under compressive loading and creep, resulting in reduced fuel leaks and stable FC performance. The C-F bond provides the stability of fluoropolymers due to the high electronegativity of fluorine atoms and high bond dissociation energy. The high content of fluorine in PTFE, PVDF and copolymers of various compositions provides a chemical resistivity to many aggressive media during prolonged heating at high temperatures, incombustibility and hydrophobicity. The main characteristics of fluoropolymers are presented in Table 1.

Two main representatives of FPs are homopolymers polytetrafluoroethylene (PTFE) and polyvinylidene difluoride (PVDF), with fluorine mass fractions of 0.76 and 0.594, respectively. The polymer density correlates with the mass fraction of fluorine, reaching the maximum for PTFE (2.12 g/cm^3^), and the lowest values are for PVDF (1.78 g/cm^3^) (Table 1). PTFE has the highest decomposition temperature (more than 415 °C) among the considered FPs. PTFE and PVDF are widely used in various fields, ranging from microelectronics to aerospace. Comprehensive overviews of the synthesis methods, properties, and existing copolymers of PTFE and PVDF are widely presented in the literature [19,20,21,22,23].

According to [24,25], non-standard form of PTFE composed of ultrafine particles with a size of ~0.6 μm with the trademark FORUM^®^, can be obtained. The morphology of ultra-dispersed PTFE is related to the fact that during the synthesis of ultra-dispersed modification, the condensation of the microspheres consisting of weakly connected films with a thickness of 5–10 nm occurs. The ultra-dispersed form is characterized by a high melting temperature of 275 °C, excellent chemical stability and a high degree of crystallinity similar to the bulk one. The crystal structure is comprised of (-CF_2_-)_n_ chains arranged in spirals directed along the z-axis. The difference in the bulk and ultra-dispersed forms is in the degree of the disordering, consisting of the relative shift of macromolecules and the rotation of CF_2_-groups [25,26].

PVDF also exhibits a high degree of crystallinity, reaching 50–70%. Polymers can crystallize in five different modifications depending on the implementation of various chain conformations (trans- and gauche-). Non-polar (α and δ) and polar (β and γ) phases can be realized. The structure of the most commonly used polar β phase is composed of chains in the TTTT conformation and has the maximum dipole moment among all of the phases of PVDF.

Unlike homopolymers, copolymers are made of the main polymer chain with attached bulky groups that increase the degree of disordering in the macromolecule, thereby reducing the crystallinity. The variation in the symmetry of the polymeric chain as a consequence of copolymerization modifies both intramolecular and intermolecular forces resulting in changes to the thermal characteristics (melting point, decomposition and glass transition temperature), elastic properties, solubility and permeability in a wide range [20].

Copolymers F2M and F42 are PVDF-based polymers with a VDF/TFE mass ratios of components of 92.75:7.25 and 61:39, respectively. As a rule, the mass fraction of fluorine and the density of copolymers F2M and F42 change in the accordance with the mass fraction of VDF/TFE (Table 1). Fluoroplast-2M (F2M) is a primary PVDF modified through the application of a small amount of TFE. The properties of the F2M copolymer are comparable to a pure homopolymer, but it has improved melt processability, greater elasticity, and a slightly lower strength.

Fluoroelastomers are high-molecular elastic polymers obtained via the radical copolymerization of fluorine-containing monomers. Fluoroelastomer SKF-26 is a copolymer of VDF and hexafluoropropylene (HFP) with a mass ratio of 75:25 and a total fluorine weight of 66%. The copolymer can stretch beyond its original length and returns to its initial size after unloading at maximum elongation at a break, reaching values of 300%.

Unlike PTFE, the copolymers of F2M, F42, and SKF26 are soluble in ketones, dimethylformamide (DMF), and dimethyl sulfoxide (DMSO). The solubility of polymers in various solvents opens the possibility of the synthesis of thin films via the tape-casting method.

The first attempt to use a fluoropolymer as a polymer matrix for an acid salt was made for the PVDF–CsH_2_PO_4_ system [27]. It was shown that the introduction of PVDF does not affect the crystal structure and thermal properties of the salt. The conductivity of the obtained samples reached 10 mS·cm^−1^ at 270 °C. The stability of the composite at a temperature of 259 °C under an atmosphere of 30% H_2_O/Ar was maintained for at least 48 h. The combination of proton conductivity and mechanical strength revealed the optimal content of the polymer to be 30 wt.% [27]. Of the greatest interest are the membranes that have both a sufficiently high conductivity and improved mechanical strength. 

The aim of this work is a comparative analysis of the composite polymer systems based on CsH_2_PO_4_ with the additives of different fluoropolymers, such as UPTFE- and PVDF-based polymers (F2M, F42, SKF26) with high mechanical and thermal stability. The high-conductive thin-film polymer composite systems were produced through the use of different methods of obtaining small salt particles, mixing and application to a matrix. More attention is paid to the analysis of the electrotransport and mechanical properties of the compositions of the membranes (1 − x)CsH_2_PO_4_-xFPs for which the thin high-conductive films can be obtained (x = 0.15–0.25). 

## 2. Experimental

The CsH_2_PO_4_ crystals were grown from an aqueous solution of Cs_2_CO_3_ and H_3_PO_4_ with a molar ratio of 1:2. CsH_2_PO_4_ particles with sizes of 1–5 µm were precipitated via the anti-solvent technique using isopropanol.

Composite electrolytes with soluble fluoropolymers (F42, F2M, and SKF26) were synthesized from a suspension of CsH_2_PO_4_ particles in a polymer solution with further drying and uniaxial pressing at P~300 MPa (x ≤ 0.15) or via the tape-casting method (for x ≥ 0.15). A small amount of polymer additive (x < 0.15) is not enough to form a strong thin-film membrane. The solvents with high boiling points (DMF), low boiling points (acetone), and their mixtures were used for the synthesis of the membranes. The homogenized suspension was spread onto a fluoroplastic substrate using an automatic tape casting machine TOB-VFC-150 (Xiamen, China). The gap height was 150–300 µm, depending on the number of layers applied. The thickness of the membranes obtained via tape casting after drying was 50–150 μm. For UPTFE polymer, the solid-state method with a further hot-pressing at T = 110–140 °C, P~100 MPa was used due to the insolubility of the polymer in the common solvents.

The proton conductivity of the membranes was measured using electrochemical impedance spectroscopy in a wide frequency range using the impedance meter P5X (1–0.5 MHz, Electrochemical Instruments, Chernogolovka, Russia) and Instek (12 Hz–200 kHz, Daejeon, Republic of Korea). The temperature dependencies of the conductivity are presented in the cooling mode. To prevent acid salt dehydration during the measurements in the high temperature range (180–245 °C), humid conditions were maintained (p_H2O_~0.3 atm). The water vapor pressure was obtained by bubbling dry argon at a constant rate (50 mL/min) through the water at T = 70 °C.

Vickers microhardness test for the tablets and the tensile strength measurements for thin-film membranes have been performed. Vickers microhardness test for uniaxially pressed tablets (5 mm in diameter and 1 mm thickness) with a relative density of 91–99% was undertaken using a Microhardness tester DuraScan 50 (EMCO-TEST, Kuchl, Austria). The load was 0.5 kgf (4.9 N) with an application time of 10 s. The values of the Vickers Hardness (HV) were obtained via the division of the load applied by the area of the impression of the diamond pyramidal indenter.

For the preparation of the samples for tensile strength testing, a punching die of a certain size was used to obtain the samples in the form of a double blade with a 5 mm × 20 mm working area. The membrane was stretched at a constant rate of 5 mm/min, and the applied load and elongation were recorded. The measurements were performed on an Instron 5944 mechanical testing machine.

The phase composition of the membranes was analyzed using X-ray diffraction using a Bruker D8 Advance diffractometer (Cu_Kα_ radiation) with a one-dimensional Lynx-Eye detector and K_β_ filter. The evaluation of the unit cell parameters and the particle size of CsH_2_PO_4_ has been carried out using the PXRD data by Topas 4.2 software (Bruker AXS, Karlsruhe, Germany) [28].

The cross-sectional morphology of the membranes was determined via the use of scanning electron microscopy Hitachi TM 1000 (Tokyo, Japan). The membranes were preliminarily coated with gold sputtering to obtain a conductive surface. The FTIR spectra in the attenuated total reflectance (ATR) mode were recorded on a Bruker Tensor 27 Spectrometer.

## 3. Results and Discussion

The composition range for high conductive membranes of (1 − x)CsH_2_PO_4_-xFPs was limited by x ≤ 0.3. Note that the compositions with x = 0.3 correspond to the different volume fractions of the polymers due to the growth of its density in the PVDF < F2M < F42 < PTFE sequence. With the further x increase, the proton conductivity decreases sharply owing to the blocking of the proton transfer pathways due to the excess amount of polymer, resulting in «conductor–insulator» percolation effect. 

**Figure 1:**
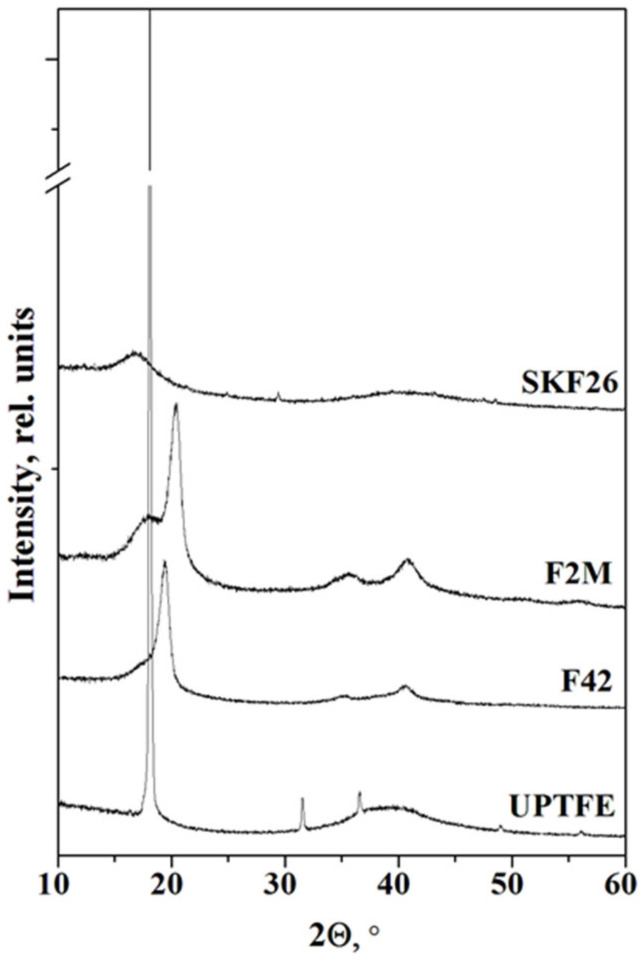
PXRD data of different fluoropolymers.

The experimental PXRD pattern for CsH_2_PO_4_ completely coincides with the calculated positions of the Bragg reflections, in accordance with [29]. The positions of the main reflexes for fluoropolymers are in good agreement with the literature data (Figure 1). The diffraction pattern of UPTFE powder is characterized by an intensive peak assigned to a crystalline phase (~18°) and the region of diffuse scattering observed at ~39° [26]. The crystal structures of VDF/TFE copolymers of different compositions have been discussed in [30]. The main diffraction peaks of copolymers correspond to the slightly shifted positions of the PVDF β-phase. The XRD pattern for fluoroelastomer SKF26 consists of only an amorphous halo at 2Θ~17° due to the low crystallinity owing to bulky HFP parts in a VDF structure.

**Figure 2:**
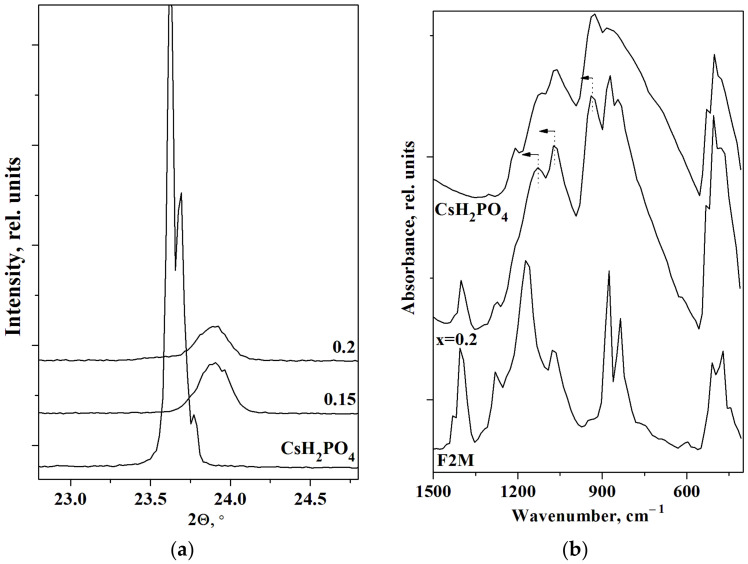
PXRD data of the (1 − x)CsH_2_PO_4_-xF2M membranes (**a**) and FTIR spectra of CsH_2_PO_4_, F2M and 0.8CsH_2_PO_4_-0.2F2M (**b**) (arrows show the direction of the a.b. displacement).

It was shown that the P2_1_/m structure of the acid salt was retained in the composite electrolytes with different FPs (UPTFE, F42, SKF26, and PVDF) [27,31,32,33]. A more intensive peak in relation to the polymer can be observed on the PXRD patterns only at x ≥ 0.15 for the membranes based on FPs with a sufficiently high degree of crystallinity. There is no chemical reaction between the components in the polymer systems, and only a slight deviation in the 2θ peak positions was observed for the composites with UPTFE and F2M due to a decrease in the parameters of the crystal cell. In addition, the intensity of the reflexes decreases more significantly than the mass fraction of CsH_2_PO_4_ in the polymer systems CsH_2_PO_4_-F-2M and UPTFE. The unusual morphology and rather high surface area of the UPTFE resulted in the disordering and amorphization of the salt [31]. While for CsH_2_PO_4_-F2M membranes, dispersion due to the insignificant interface interaction takes place. Figure 2a demonstrates the displacement of the main reflections (011 and −111) of the CsH_2_PO_4_ phase in the composites with F2M and their decrease. The reflections in the polymer composites move toward the larger angles due to a slight decrease in the unit cell parameters of CsH_2_PO_4_ in the volume of the polymer matrix.

The FTIR spectra of the polymer electrolytes are similar to the pure CsH_2_PO_4_ (Figure 2b). The range of 1500–500 cm^−1^ corresponds to the main characteristic absorption bands of F2M, and 1300–500 cm^−1^ is related mainly to the spectral range of νPO_4_ tetrahedra. Note that the FTIR spectrum at x = 0.2 is the superposition of the absorption bands of CsH_2_PO_4_ and F2M. For x = 0.2, the additional absorption bands in the range of 1400, 1274, 875, 835, and 509 cm^−1^ appear to be the FTIR spectrum of CsH_2_PO_4_. The shift in the absorption bands of 930 and 1122 cm^−1^ of CsH_2_PO_4_ in the composite to the 939 and 1133 cm^−1^ (marked in Figure 2b) is related to the insignificant strengthening of the P-O bonds in the polymer membranes. Both the FTIR and XRD data are consistent and confirm the insignificant CsH_2_PO_4_ changes due to the negligible interface interaction and salt dispersion.

**Figure 3:**
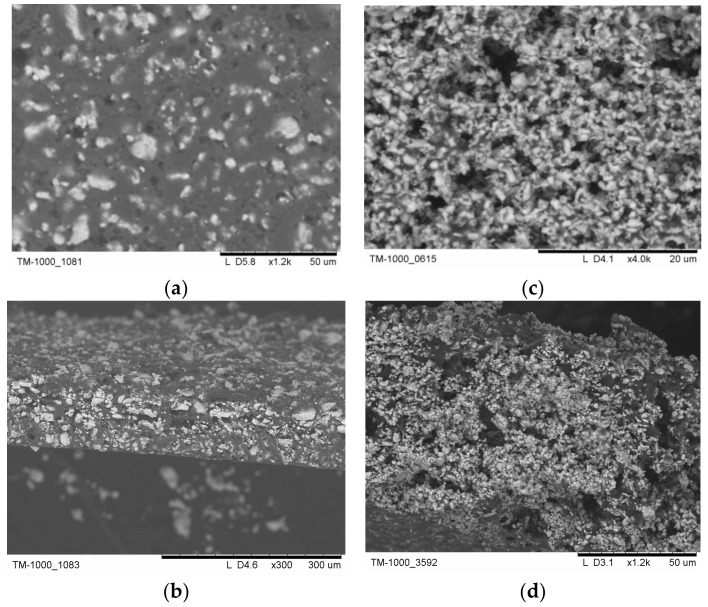
SEM images of the top view (**a**,**c**) and cross-section (**b**,**d**) of thin films x = 0.2 (F42) with the salt particles precipitated using the anti-solvent technique with isopropanol (**a**,**b**); film obtained from the suspension of CsH_2_PO_4_ in SKF26 polymer solution pretreated in a bead mill (**c**,**d**).

As a rule, membrane thickness and mechanical characteristics can be limited by the particle size of the inorganic component and the uniformity of its distribution. SEM images (Figure 3) present the differences in the morphology of the membranes with the salt particles obtained via the use of various methods. The thickness of the membranes obtained via tape casting was 50–100 μm (Figure 3b,d). Note that durable thin films were obtained for the compositions of x ≥ 0.15. Thin films with mass fractions of F42, x = 0.2, with salt particles precipitated through the anti-solvent technique using isopropanol are characterized by different sizes in terms of the salt particles, with sizes of 1–5 μm, resulting in less even distribution (Figure 3a). Thin films with x = 0.2 obtained from the suspension of CsH_2_PO_4_ in the SKF26 polymer solution pretreated in a bead mill are characterized by more uniform distribution and a smaller size in terms of the salt particles (~300 nm, Figure 3c,d).

**Figure 4:**
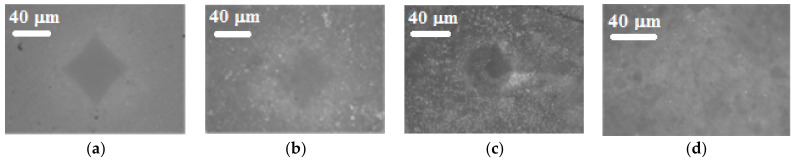
The microscope images of the indenter impression on the membrane surface of CsH_2_PO_4_ (**a**) and 0.85CsH_2_PO_4_-0.15FP composites with UPTFE (**b**), F42 (**c**), and SKF26 (**d**).

The microhardness test for the CsH_2_PO_4_-FPs polymer composites was used to evaluate the mechanical characteristics of the sample in the form of the tablet for x ≤ 0.15 [34] (Figure 4). The microhardness value for the tablet of the pressed polycrystalline CsH_2_PO_4_ was HV~34 (333.4 MPa), whereas for the composite polymer membrane, the size of the indenter impression on the membrane surface decreases, resulting in lower values in terms of microhardness (Figure 4). The HV for F42- and UPTFE-based membranes have close values of 11.8 and 12, respectively. For the membranes with SKF-26 (x = 0.15), there was no visible impression of the indenter. It was quickly erased due to the elastomeric nature of the polymer and, as a consequence, a short recovery time for the membrane surface. It confirms that polymer composite membranes can resist plastic deformation and that their mechanical properties are much higher than of the initial CsH_2_PO_4_ salt.

**Figure 5:**
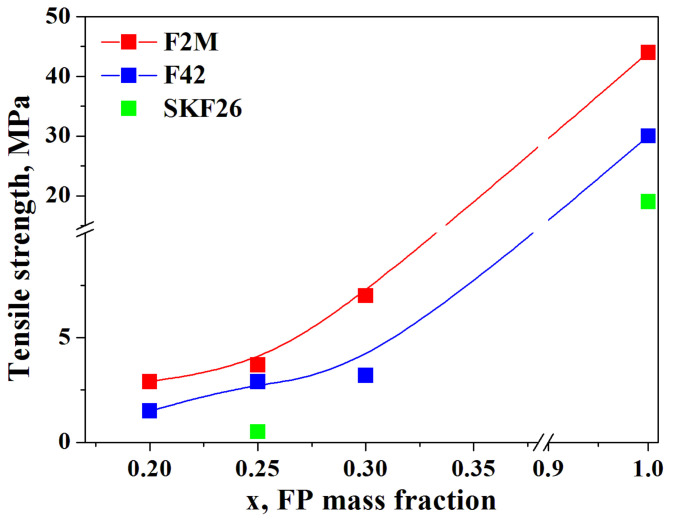
The tensile strength of the thin-film CsH_2_PO_4_-based membranes with F2M, F42, and SKF26 in comparison with the initial FPs.

According to Table 1, the F2M copolymer is characterized by the highest value of tensile strength (34.3–55 MPa) among the FPs considered. The same tendency was observed for CsH_2_PO_4_-FPs polymer composites. Figure 5 presents the values of the tensile strength of the thin-film membranes in comparison with the literature data of the initial FPs. A maximum value of 7 MPa was obtained for the 0.7CsH_2_PO_4_-0.3F2M membrane. The tensile strength values for 0.75CsH_2_PO_4_-0.25SKF26 were rather low and did not exceed 1 MPa; however, the highest elongation before the break of 170% was observed due to the elastomeric properties of the polymer.

Thus, the mechanical properties of the obtained proton membranes are characterized by high values comparable to the best polymer membranes. 

Obviously, the size and shape of the particles, and their distribution in the membrane, influence both the mechanical strength and the values of proton conductivity. As for CsH_2_PO_4_, the difference in the morphology of its particles does not affect the proton conductivity since the surface conductivity for acid salt is comparable to the bulk one. At the same time, the polymer membranes with different aspect ratios of salt (length to diameter) provide a different number of CsH_2_PO_4_ contacts in the volume of the matrix, which result in more developed pathways for proton transfer. For example, the proton conductivity of (1 − x)CsH_2_PO_4_-xSKF26 membranes with the platelet salt particles precipitated from the water solution via the anti-solvent technique using ethyl alcohol in the presence of ethylene glycol was ~1 order of magnitude lower in comparison with small (about 300–500 nm) spherical particles obtained via the ball-milling process. Obviously, the small particles with near-spherical shapes are the most prominent for the transport characteristics of the polymer composite membranes due to the maximum number of salt contacts [33].

**Figure 6:**
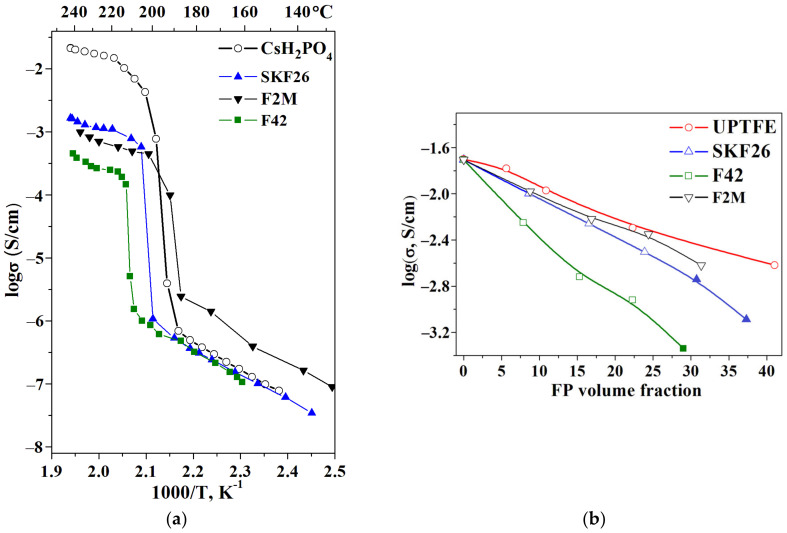
Temperature dependencies of the proton conductivity of the thin-film membranes with different soluble FPs: SKF26, F42, F2M, x = 0.2 (**a**); Isotherms of the proton conductivity of CsH_2_PO_4_-FPs as a function of FPs volume fraction at T = 240 °C (**b**) (filled symbols: thin-film membrane; open symbols: tablet).

Temperature dependence of the proton conductivity of the composite polymer membranes is similar to the initial salt and can be characterized by the low- and high-temperature regions (Figure 6a). In the high-temperature region, CsH_2_PO_4_ exists in the superionic phase, with the high proton conductivity reaching a value higher than ~3 × 10^−2^ S/cm. The observed jump in the proton conductivity of CsH_2_PO_4_ is due to the fact that the superproton phase transition is four orders of magnitude greater. The conductivity dependence for the low-temperature region (from the room temperature to the superionic phase transition) has an Arrhenius character with an activation energy of 0.8 eV. The activation energy of the conductivity of the polymer membranes in the high-temperature phase is about 0.4 eV, similar to CsH_2_PO_4_. In the polymer composite electrolytes, a decrease in the proton conductivity in the high-temperature region close to linear is observed (Figure 6a). The low-temperature conductivity of SKF26 and F42 containing thin-film membranes is close to pure CsH_2_PO_4_. At the same time, the conductivity of the CsH_2_PO_4_-F2M polymer system is 0.5–1 order of magnitude higher than CsH_2_PO_4_ due to the interface interaction and salt dispersion. With the further x increase, the conductivity decreases both in the high- and low-temperature regions of the polymer composite electrolytes. The decrease in the proton conductivity in the high-temperature region (Figure 6a) is more significant due to the differences in the conductivity at higher temperatures. Slightly higher conductivity values for the CsH_2_PO_4_-UPTFE membrane can be due to another way of the synthesis that excludes the formation of a continuous polymer film on the surface of the salt particles. The conductivity jumps due to the superproton transition to the monoclinic (P2_1_/m) phase, which shifts to lower temperatures of more than 20 °C for the membranes of CsH_2_PO_4_-F2M due to the more pronounced interface interaction. The interface interaction with the negligible strengthening of the P-O bonds determines the shift of the phase transition temperature and the expansion of the range of CsH_2_PO_4_ superproton phase existence in the polymer electrolytes, which is important and a possible perspective in terms of SAFCs. The close values of proton conductivity in the high-temperature phase were obtained for the composite systems based on the CsH_2_PO_4_ and PVDF homopolymer [29]. However, the effect of shifting the temperature of the superionic phase transition and the extension of the superionic phase range was not observed. 

The main tendency of the proton conductivity dependencies with different FP volume fractions can be tracked for the tablet samples in the range up to 0.15 (f_V_ = 0.20–0.25) (Figure 6b). For all of the polymer systems except UPTFE, the samples for the conductivity measurements were obtained via the same method. According to Figure 6, the change in the conductivity is close to linear. The selected FPs are dielectrics (Table 1), and the addition of these polymers will inevitably lead to a decrease in the proton conductivity of the composite membranes due to the percolation effect of the “conductor-insulator”. The polymer layer with sufficient thickness on the surface of the salt particles acts as a barrier for proton transport. The polymer systems based on F2M, UPTFE and SKF26 show close and high conductivity values.

Despite the closed nature of fluoropolymers, they have some processing peculiarities. The temperature of the melting for F42 and F2M copolymers is preceded by the temperature of the decomposition. However, at 150–180 °C, only the crystallites melt, and the polymer does not pass into a viscous flow state up to the decomposition. Some softening of the polymer may contribute to a better coating of the surface of the salt particles with the polymer. 

The boiling temperature of the solvent used can influence the morphology of the membranes. For the films cast with the low-boiling point solvent (acetone), the pores may be observed due to the high evaporation rate of the solvent. Therefore, the high-boiling solvent DMF or the mixtures of DMF and acetone are preferable for obtaining thin-film proton membranes via the tape-casting technique. Another way to obtain denser thin-film proton membranes is pressing the membranes. This is a simple and convenient way to densify the membranes of F42 and F2M polymers; however, this method is not suitable for SKF26 membranes due to their elastomeric properties. Probably the absence of the phase transitions up to the decomposition temperature also affects the properties of the SKF26-containing membranes.

**Figure 7:**
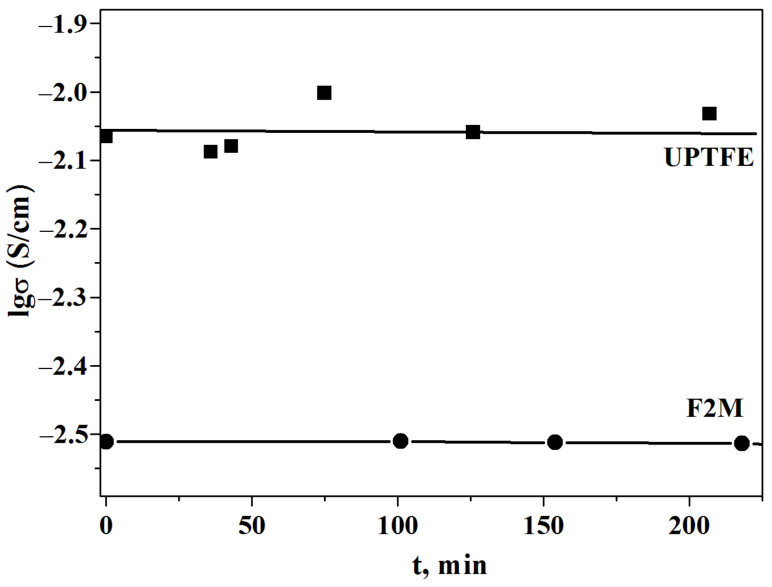
The dependence of the conductivity of 0.85CsH_2_PO_4_-0.15UPTFE and 0.8CsH_2_PO_4_-0.2F2M under the term storage at T = 238 °C P_H2O_~0.3 atm.

Despite the decrease in conductivity, the polymer membranes are chemically and thermally stable. Long-term thermal storage of the membranes revealed the stability of the proton conductivity at T = 238 °C and p_H2O_~0.3 atm (Figure 7).

Thus, there is a significant improvement in the mechanical properties of the polymer membranes and the synthesis of thin films (50–100 μm) despite the decrease in proton conductivity. Even small concentrations of FPs can improve the mechanical properties of CsH_2_PO_4_ membranes and increase their hydrophobicity and stability in a humid environment, which is very important for SAFCs and other acid–salt applications. At the same time, higher FP concentrations create the prospect of the synthesis of thin-film membranes via the tape casting method. Additionally, sequential layer-by-layer deposition can be used for a more reliable connection of the components for the dense thin films for both proton membranes and membrane–electrode assemblies.

## 4. Conclusions

A detailed study and comparison of the proton conductivity, structural properties, mechanical characteristics, and morphology of the (1 − x)CsH_2_PO_4_-xFPs systems (FP = PTFE, F2M, F42, and SKF26) were carried out. The used fluoropolymers are shown to be chemically inert matrices for CsH_2_PO_4_. CsH_2_PO_4_-based composite membranes with a thickness of ~50–100 μm and uniform distribution of the salt particles in the volume of polymer matrix were obtained for the soluble fluoropolymers (F2M, F42, and SKF26). The influence of the size and the morphology of CsH_2_PO_4_ particles in the polymer composite systems on the proton conductivity have been studied. The different methods used to obtain polymer membranes were carried out and tested. The analysis of the optimal conditions for the obtained polymer compositions was made. Evenly distributed systems obtained in a bead mill have an advantage. Proton membranes are characterized by a decrease in superion conductivity in comparison with the initial salt and significantly increased mechanical strength. The optimal combination of the conductivity, mechanical strength and hydrophobic properties of (1 − x)CsH_2_PO_4_-x fluoropolymer permit the creation of promising compositions of proton-conducting membranes (x = 0.15–0.25) for use in fuel cells. Further ways to improve the properties of high-conductive thin-film membranes are being considered.

## Figures and Tables

**Table 1:** Composition, thermal and the mechanical and electrical properties of fluoropolymers.

Trademarks: Russian/Foreign	Chemical Formula	Thermal Properties	Specific Volume Electroresistance, Ohm·cm	Tensile Strength, MPa	Density, g/cm^3^
FORUM^®^/ UPTFE	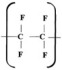	T_m_ = 275 °C T_dec_~415 °C	10^12^–10^13^	14.7–33	2.12–2.2
F42/ Kynar 7200	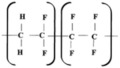	T_m_~150–160 °C T_dec_~360 °C	10^7^–10^8^	14.6–45.1	1.9–2.0
F2M/ Kynar	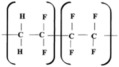	T_m_ = 177 °C T_dec_~350 °C	10^8^–10^9^	34.3–55	1.78
SKF26/ Viton A	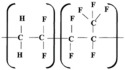	T_m_ = 250 °C T_dec_~320 °C	10^11^	17.6–20.6	1.83

Where T_m_: temperature of melting; T_dec_: temperature of decomposition.

## Data Availability

Not applicable.
